# How [18F]-FDG-PET/CT Affects Clinical Management of Patients with Germ Cell Tumors in the Real World

**DOI:** 10.3390/cancers15143652

**Published:** 2023-07-17

**Authors:** Cecilia Liang, Julia Sekler, Brigitte Gückel, Christina Pfannenberg, Helmut Dittmann, Ferdinand Seith, Bastian Amend, Konstantin Nikolaou, Christian Philipp Reinert

**Affiliations:** 1 Diagnostic and Interventional Radiology, Department of Radiology, University Hospital of Tuebingen, Hoppe-Seyler-Str. 3, 72076 Tübingen, Germany; 2 Nuclear Medicine and Clinical Molecular Imaging, Department of Radiology, University Hospital of Tuebingen, Otfried-Mueller-Strasse 14, 72076 Tübingen, Germany; 3 Department of Urology, Tübingen University Hospital, 72076 Tübingen, Germany; 4 Cluster of Excellence iFIT (EXC 2180) “Image Guided and Functionally Instructed Tumor Therapies”, University of Tübingen, 72076 Tübingen, Germany; 5 German Cancer Consortium (DKTK), Partner Site Tübingen, 72076 Tübingen, Germany

**Keywords:** PET/CT, germ cell tumors, seminoma, clinical management

## Abstract

**Simple Summary:**

This study aimed to assess the impact of PET/CT on the management of patients with germ cell tumors (GCTs) in a real-world setting, specifically in terms of avoiding invasive procedures, reducing additional diagnostic imaging, and influencing treatment decisions. A total of 43 male GCT patients were included in this study, and their intended management before and after PET/CT was documented. The results showed that PET/CT caused changes in oncologic staging in 51% of patients, with 16% of patients upstaged, 23% of patients downstaged, and 11% of patients experiencing cancer relapse. Although the number of patients receiving curative treatment remained stable, there were notable changes in the intended therapeutic interventions. Planned chemotherapy increased from three to eleven patients, while planned surgical resection decreased from eleven to two patients. Moreover, PET/CT helped to avoid invasive procedures in 19% of cases and to reduce the need for additional diagnostic procedures in 58% of cases. In conclusion, the use of PET/CT had a significant impact on the clinical stage, resulting in a reduction in invasive and diagnostic procedures. These findings are expected to have even greater significance in the future as treatment options improve and GCT patient survival rates increase.

**Abstract:**

Objective: The aim of this study was to evaluate the impact of PET/CT on clinical management of patients with germ cell tumors (GCTs) conducted in a real-world setting, including avoidance of invasive procedures, additional diagnostic imaging, and changes in treatment. Methods: Patients with GCTs were prospectively enrolled into a PET/CT registry study between May 2013 and April 2021. Intended patient management prior and after PET/CT was documented using standardized questionnaires. Changes in oncologic staging and clinical management after PET/CT were recorded, including planned treatment and planned additional diagnostics. Results: Forty-three male patients with GCTs were included consecutively in this study. After PET/CT, oncologic staging changed in 22/43 patients (51%), with upstaging in seven cases (16%), downstaging in ten cases (23%), and cancer relapse in five cases (11%). The number of patients with intended curative treatment remained stable, while a considerable change in intended therapeutic intervention was noted after PET/CT, with an increase in planned chemotherapy from three to eleven patients and a decrease in planned surgical resection from eleven to two patients. In addition, PET/CT contributed to preventing patients from intended invasive procedures including biopsy and surgery in 8/43 (19%) cases and from additional diagnostic procedures in 25 (58%) cases. Conclusion: With the use of FDG-PET/CT as a tool to guide patient management in GCTs, we observed a notable impact on clinical staging and a consequent reduction in the need for additional invasive and diagnostic procedures. These findings are expected to be even more consequential in the future as treatment modalities improve and the life expectancy of GCT patients further increases. Key Points: PET/CT considerably influences the clinical stage of GCT patients. PET/CT has remarkable influence on the choice of therapeutic interventions and reduces additional diagnostic procedures.

## 1. Introduction

Germ-cell tumors (GCTs) account for only about 1% of all cancers in men. Nevertheless, they are among the most common cancers in younger men between the ages of 15 and 40 and their numbers are steadily increasing. Diagnosis is based on histology, and 55–60% of GCTs are pure seminomas, while 40–45% of GCTs are non-seminomas. In addition, serum tumor markers are determined as standard, including α-fetoprotein (AFP), human chorionic gonadotropin beta subunit (β-hCG), and lactate dehydrogenase (LDH), although studies have shown low sensitivity for detection of cancer relapse [1]. Contrast-enhanced computed tomography (CT) of the chest, abdomen, and pelvis is recommended for detection of nodal and distant metastases. CT usually provides good image quality in the retroperitoneal and mediastinal lymph nodes areas, but in lean young men the delineation of lymph nodes from surrounding tissue may be difficult. Furthermore, the accuracy of CT can be compromised by false-negative findings, particularly because it is unable to detect the presence of disease in normal-sized lymph nodes and fails to identify a viable tumor in residual masses after therapy [2].

[18F]fluorodeoxyglucose (FDG) PET/CT has been shown to be highly accurate in detecting both the primary tumor and metastases in many malignant solid tumors (e.g., lung, colon, breast). In GCTs, there is currently limited evidence for the use of [18F]fluorodeoxyglucose (FDG) positron emission tomography (PET)/CT for primary staging in GCTs.

Data for FDG-PET/CT in detection of GCT relapse, from a systematic review with a meta-analysis, indicate a sensitivity of 78%, a specificity of 86%, and an accuracy of 84% for seminoma after chemotherapy [3]; however, in patients with non-seminomatous GCTs after chemotherapy, the sensitivity of PET/CT is only 40%. This and the high rate of false-negative findings due to lack of tracer uptake of teratomatous areas in the residual tumor limit the validity of FDG-PET/CT for non-seminoma. Current EAU guidelines and a previous study [4] recommend the use of PET/CT in seminomas for classification of residual masses greater than 3 cm after chemotherapy, whereas there is no recommendation for primary staging or restaging with PET/CT for non-seminomas [5,6]. In clinical practice, imaging techniques are not always used in accordance with the active surveillance protocol, with patients receiving both under- and overuse of diagnostic imaging [7]. Despite current evidence-based recommendations, [18F]FDG-PET/CT is commonly used and recommended in clinical practice evaluation of treatment response and residual masses in GCT patients [5]. Several studies have demonstrated the high diagnostic accuracy of PET/CT for staging and restaging [8,9,10]. In addition, PET/CT is believed to prevent unnecessary treatment such as additional diagnostics and therapy [11]. This is particularly relevant in view of the growing evidence that survivors experience significant long-term morbidity and impaired health-related quality of life associated with treatment [12,13].

Therefore, the aim of this study was to evaluate the impact of PET/CT on clinical management of GCT patients conducted in a real-world setting, including avoidance of invasive procedures such as biopsies and surgery, as well as avoidance of additional diagnostic imaging and changes in treatment.

## 2. Methods

This study was based on a prospective PET/CT registry with a total of 7373 patients [14]. This study was reviewed and approved by the local ethics committee. Informed consent was obtained from all patients to use the data for research.

### 2.1. Patient Cohort

The cohort included all patients with GCTs who were enrolled in the PET/CT registry between May 2013 and April 2021 for primary staging, restaging, or suspected relapse. For patients with repeated PET/CT, only the first scan was considered, unless previous PET/CT scans were performed at external hospitals. Information on imaging prior to PET/CT, histological examinations, and follow-up was obtained from patient records.

### 2.2. PET/CT Imaging

All PET/CT scans were conducted using a state-of-the-art clinical scanner Biograph mCT^®^ from Siemens Healthineers in Erlangen, Germany. Prior to the [18F]FDG-PET/CT examination, patients fasted overnight. The PET/CT scan began 60 min after the administration of weight-adjusted 300–350 MBq [18F]FDG through intravenous injection. CT scans were performed using weight-adjusted 90–120 mL intravenous CT contrast medium (Ultravist 370 from Schering AG, Berlin, Germany) if there were no contraindications, otherwise, the contrast medium was not used. PET imaging was acquired from the skull base to the thigh, covering six to eight bed positions, and reconstructed using a 3D ordered subset expectation maximization algorithm with two iterations, 21 subsets, a Gaussian filter of 2.0 mm, a matrix size of 400 × 400, and a slice thickness of 2.0 mm. The acquisition time for PET was 2 min per bed position.

### 2.3. Patient Management

In the registry study, referring physicians completed standardized questionnaires before and after PET/CT according to the concept of the “National Oncologic PET Registry” (NOPR) on intended patient management. Details are described in the publications of Hillner et al. [15] and Pfannenberg et al. [14]. The questionnaire focused on questions regarding the specific PET/CT indication, the intended treatment concept, and the intended diagnostic imaging including invasive diagnostic procedures if PET/CT had not been available. The same questions had to be answered again after notification of the PET/CT findings.

### 2.4. Data Analysis

Histologic diagnosis and clinical stage were obtained from clinical reports. Information on the number and location of metastases was extracted from the PET/CT reports.

Intended patient management was evaluated before and after PET/CT, considering the following changes:

- Therapy, i.e., changes in intended treatment goals (curative, palliative) and intended treatment modalities (e.g., chemotherapy, surgery, and radiotherapy).
- Diagnostic procedures. i.e., changes in intended diagnostic procedures (e.g., biopsy and additional imaging).

Overall survival and follow-up time were assessed using available patient records.

### 2.5. Statistical Analysis

All statistical analyses and graphical illustrations were conducted using SPSS version 24 (IBM Corporation, Armonk, NY, USA) and open-source software (SankeyMATIC, https://sankeymatic.com, accessed on 21 May 2023).

To examine changes in management, we initially categorized management plans into specific categories (detailed in Table 4) and compared them before and after PET/CT. The analysis primarily focused on changes related to treatment concept, additional invasive diagnostics, and imaging. Results were reported as absolute and percentage frequencies. Sankey graphs were used to visualize changes in the clinical stage and frequency of transitions from the pre-PET/CT to the post-PET/CT management plan.

## 3. Results

### 3.1. Patient Cohort

**Figure 1:**
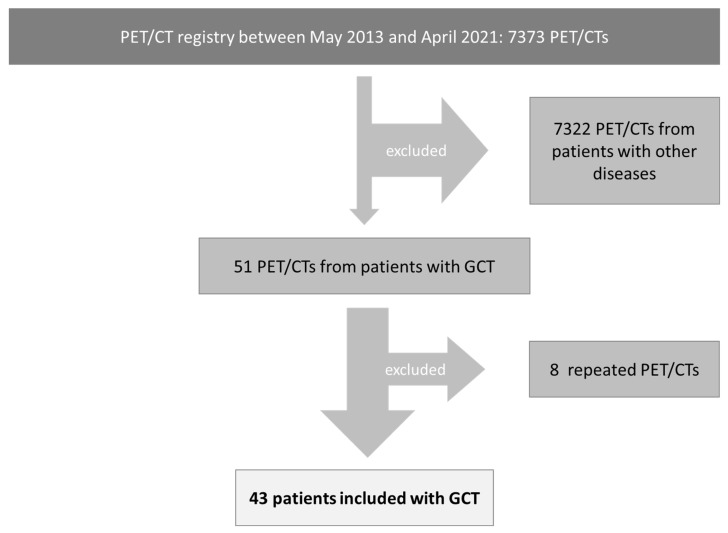
Inclusion criteria.

In total, 51 PET/CTs of 7373 PET/CTs registered between May 2013 and April 2021 were registered with GCTs. Eight PET/CTs were repeated examinations and were excluded, resulting in the inclusion of 43 patients (all male, mean age 44 ± 10 years) with GCTs, who underwent [18F]FDG-PET/CT (see Figure 1). Testicular GCTs were diagnosed in 39 patients including thirty (74%) patients with seminoma and nine (21%) patients with non-seminomatous GCTs. Two (5%) patients were diagnosed with extratesticular GCTs, exclusively mediastinal seminoma. In two patients, the histopathological diagnosis could not be assessed. All patients underwent surgical resection of the primary tumor before PET/CT. The clinical stage before PET/CT was available for 41 patients. Clinical stage I was documented in nine patients (16%), clinical stage II in sixteen patients (37%), and clinical stage III in thirteen patients (30%). The clinical stage was not documented in two patients before PET/CT. Cross-sectional imaging including CT (29 patients, 67%) and MRI (one patient, 2%) was performed before PET/CT in the majority of patients. In ten patients (23%), no previous imaging was documented. The mean number of days between previous imaging and PET/CT was 290 days (9–4578 days). Patient death was noted in three patients. Patients’ characteristics are listed in Table 1.

### 3.2. Indications and Findings of PET/CT

PET/CT was utilized for initial staging in two patients, while the majority of patients (28 patients, 65%) underwent PET/CT for restaging purposes, and 13 patients (30.2%) for dignity assessment. Further information is provided in Table 2.

The most frequent GCT manifestations were found in lymph nodes (36/43, 84%), followed by visceral organ lesions (4/43, 9%), pulmonary lesions (3/43, 7%), bone lesions (1/43, 2%) and brain lesions (1/43, 2%). Suspicious lesions could be specified as metastases or relapse, mainly in lymph nodes (11/43, 26%), followed by visceral lesions (2/43, 5%), pulmonary lesions (2/43, 5%), bone lesions (1/43, 2%), and brain lesions (1/43, 2%). Several suspicious lesions could be classified as non-metastatic, mostly lymph nodes (7/43, 16%), leading to downstaging. In addition, PET/CT led to a new secondary diagnosis in four cases including a suspicious prostate lesion, sarcoidosis, esophagitis, and retroperitoneal liponecrosis. The PET/CT findings are summarized in Table 3.

**Figure 2:**
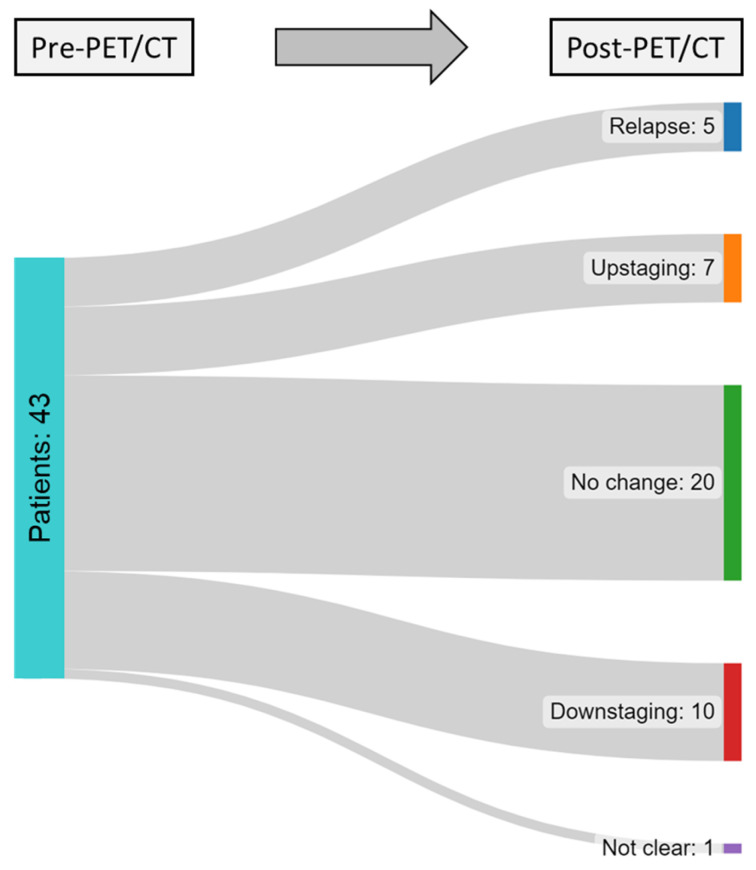
Alterations in the clinical stage of patients with GCTs following PET/CT examination.

**Figure 3:**
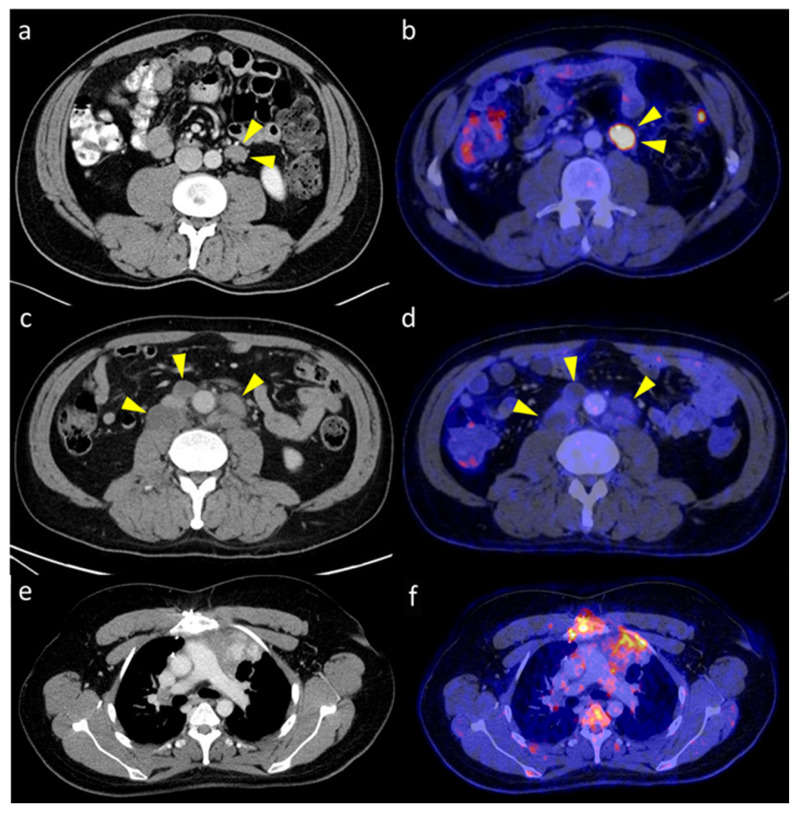
[18F]FDG PET/CT and additional CT of patients with GCTs: (**a**,**b**) A patient with testicular seminoma and suspicious para-aortal lymph node on CT showing increased FDG uptake on PET/CT, leading to upstaging; (**c**,**d**) a patient with testicular seminoma and suspicious retroperitoneal lymph nodes on CT that appears without FDG uptake on PET/CT, resulting in downstaging; (**e**,**f**) a patient with mediastinal seminoma and residual mediastinal mass on CT that was found to be recurrent on PET/CT.

Table 4 and Figure 2 demonstrate the impact of the PET/CT on clinical staging of patients with GCTs. Overall, PET/CT resulted in upstaging in seven patients (16%), downstaging in ten patients (23%), and relapse in five patients (12%). In 20 patients (47%), PET/CT had no impact on the clinical stage. In one patient, the state was indicated as indefinite by referring physicians. Figure 3 shows examples of patients in whom PET/CT contributed to a change in clinical stage.

### 3.3. Impact of PET/CT on Clinical Patient Management

**Figure 4:**
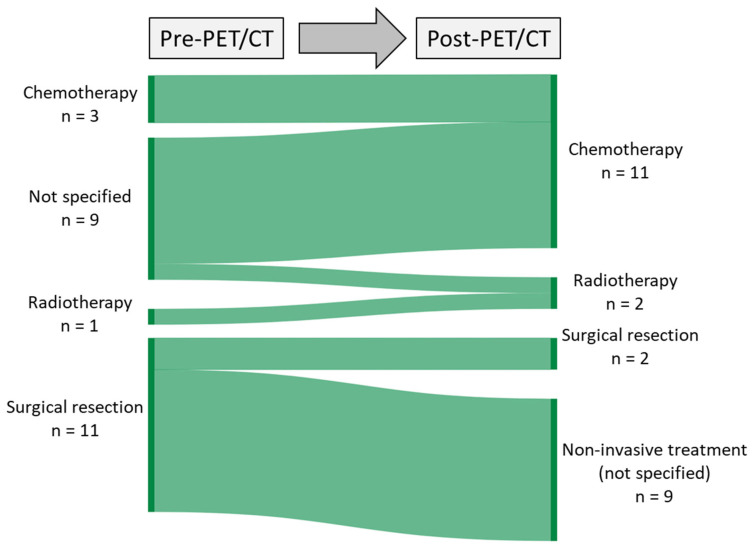
Sankey diagram depicting the alterations in the proposed treatment approach pre and post PET/CT scan, considering the input from referring physicians.

The overall number of patients with intended curative treatment remained stable (26 patients, 61%), whereas two cases with palliative treatment were added in the questionnaires after PET/CT. The pre- and post-PET/CT questionnaires showed a change in intended therapeutic procedures, with an increase in intended chemotherapy and a decrease in intended surgical resection after PET/CT (Figure 4).

**Figure 5:**
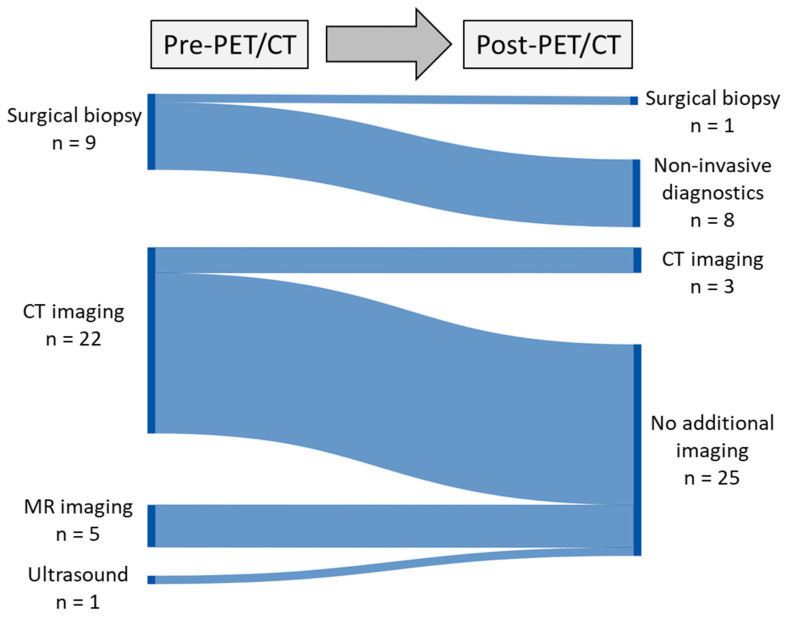
Sankey diagram depicting the alterations in intended additional imaging and invasive diagnostic procedures pre and post PET/CT scan, considering the input from referring physicians.

In addition, both the number of intended invasive diagnostic procedures, including surgical biopsy, and the number of additional imaging procedures decreased considerably after PET/CT (Figure 5).

Table 5 summarizes the influence of PET/CT on the intended clinical management of patients.

## 4. Discussion

In this study, we investigated the impact of PET/CT on therapeutic and diagnostic management in a cohort of GCT patients who underwent FDG-PET/CT imaging in a real-world setting.

We observed remarkable changes in oncologic staging after PET/CT in 51% of the patient cohort, including upstaging in 16%, downstaging in 23%, and relapse in 12% of the patient cohort. PET/CT allowed specifying suspicious lesions as metastases or relapse or classifying lesions as non-metastatic, mostly in lymph nodes, which were the most common metastatic site. While surgical pathology determines the T category, imaging plays a critical role in determining the N and M components of testicular tumor staging. The higher sensitivity of PET/CT as compared to CT in detecting metastases in patients with GCTs has been described in several studies [10,11,16] and is consistent with our results. Despite the wide acceptance of PET/CT in other tumor types, the only validated and recommended PET/CT indication for testicular tumors in current guidelines is to determine residual tumor burden after chemotherapy [17], which is also limited to seminoma.

Compared to other forms of cancer [14], PET/CT only led to a change in intended curative and palliative treatment in 5% of cases (2 out of 43). This observation could be attributed to the significantly improved prognosis and survival rates of testicular cancer following the introduction of cisplatin chemotherapy.

Five years after diagnosis, the relative survival rate is approximately 96% compared to the general male population of the same age. Even after 10 years, this rate is only slightly lower (95%) [18]. Accordingly, only one tumor-related death was documented in our cohort. Moreover, GCTs primarily affect young male adults. Therefore, curative approaches are usually selected for these patients.

The analysis of the questionnaires before and after PET/CT revealed a change in intended mode of therapeutic interventions with an increase in intended chemotherapy and radiotherapy and a decrease in intended surgical resection. The intended invasive diagnostics and additional imaging were both remarkably reduced after PET/CT. Identification of patients at increased risk for additional surgical intervention is primarily based on pre-operative imaging. This is a technically demanding procedure associated with significant treatment-related morbidity [19,20]. In approximately 30% of cases, additional surgical interventions such as nephrectomy or vascular reconstruction are necessary during the procedure [21,22,23]. Conversely, adjuvant treatment strategies, such as cytostatic chemotherapy following inguinal orchiectomy, have shown excellent cure rates. However, it is crucial to utilize these treatments judiciously to prevent overtreatment and to minimize potential toxic side effects [24]. PET/CT has demonstrated its effectiveness in reducing the need for further diagnostic procedures across various cancer types [14,15,25,26,26,27,28]. Ambrosini et al. conducted a study comparing the utility of PET/CT with standard imaging in GCT patients and found that clinical management changes occurred in 21.6% of seminomas and 25.7% of non-seminomas [29]. A meta-analysis of four studies involving 130 seminoma patients revealed that PET/CT significantly reduced overtreatment rates from 72% to 30%, while undertreatment rates decreased from 14% to 7% [30]. Another meta-analysis concluded that negative PET/CT results could prevent unnecessary adjuvant therapy following chemotherapy, given its excellent negative predictive value [3].

In contrast to existing research that has evaluated the sensitivity of PET/CT [3,30], our study aims to examine the impact of PET/CT on clinical management and, beyond Ambiosini et al. also included the impact PET/CT has on prevention of diagnostic imaging and invasive procedures [29].

Among our patient cohort, a majority of 76.7% of the patients had previously undergone imaging. However, PET/CT identified new secondary diagnoses in 9.3% of patients, which is important for determining therapy and patient management. Clinically relevant secondary findings such as inflammation, vascular complications, and unknown secondary tumors are crucial to consider, in addition to the status of known or suspected disease. Incidental findings, although common, can significantly impact investigative protocols, reporting strategies, and further management, particularly in the context of oncology [31].

Metastases were found in one organ in 58.1% of the patients, whereas from two to three organs or more than three organs were affected in 16.3% of the patients. Consistent with our findings, the most common metastases of GCTs to solid organs are the lungs, followed by the liver and brain [32]. Approximately 9–11% of patients with GCTs develop atypical metastases [33]. Studies have indicated that distant lymph nodes and lung metastases are favorable prognostic factors, while bone and liver metastases are associated with poor survival outcomes in testicular cancer [34].

We acknowledge some limitations of our study. First, part of the patient cohort was treated in external hospitals and information about previous examinations and histology was limited to the information given in the questionnaires. The variable time gap between previous imaging and PET/CT as well as the absence of histological confirmation and insufficient follow-up imaging for several patients in our study may introduce potential biases and uncertainties in the diagnostic process. Second, another point that must be taken into consideration is that registry-based studies are inherently limited by their observational design, which introduces the possibility of unrecognized biases [35]. Consequently, the potential for sample biases and an overestimation of PET/CT’s impact cannot be overlooked, as patient allocation is dependent on individual physician judgments. To mitigate these limitations, it is crucial to promote standardized recommendations for PET/CT imaging, restricting its use to established clinical indications and cases where conventional imaging methods fail to provide definitive diagnostic information. Additionally, it is important to recognize that the information captured through questionnaires regarding patient management does not always align with the final treatment decision. Instead, it primarily reflects the intention for therapy or additional diagnostics at different time points.

Third, in a routine clinical setting, the process of staging and the change in clinical stage usually encompasses an interdisciplinary decision making where various clinical factors such as individual patient preferences play a role. For our analysis, however, we focused solely on the role of PET/CT and its influence on planned patient management and tumor staging. To accomplish this, we relied on the information provided in the questionnaires. Fourth, larger cohorts are needed to reliably detect statistical correlations between histopathologic differences in GCTs and the change in clinical stage or mortality. Fifth, despite the limited clinical value of PET due to relatively low FDG avidity in non-seminomas, we included these patients in our analysis. Remarkably, over 50% of these patients demonstrated a change in their clinical stage. Sixth, access to PET/CT varies worldwide due to availability and issues of reimbursement, limiting its applicability outside highly resourced settings [36].

## 5. Conclusions

With the use of FDG-PET/CT as a tool to guide patient management in GCTs, we observed a notable impact on clinical staging and a consequent reduction in the need for additional invasive and diagnostic procedures. These findings are expected to be even more consequential in the future as treatment modalities improve and the life expectancy of GCT patients further increases.

## Figures and Tables

**Table 1:** Patients’ characteristics.

	No. of Patients (n = 43)	%
**Sex**		
Male	43	100
**Age (years)**		
Median	46	
Interquartile range (25–75)	38.5–51.0	
**In-/outpatients**		
Inpatients	43	100
Outpatients	0	0
**Patient follow-up time (years)**		
Mean	4	
Minimum	0	
Maximum	9	
**Death rate**		
Documented cases of death	3	7.0
Tumor-related deaths	1	2.3
**Histological diagnosis**		
Testicular GCTs		
Seminoma	30	74.4
Non-seminoma	9	20.9
Embryonal carcinoma	4	9.3
Mixed germ cell tumor	5	11.6
Extratesticular GCTs		
Mediastinal seminoma	2	4.7
Unknown	2	4.7
**Clinical stage**		
I	9	20.9
II	18	41.9
III	14	32.6
Unknown	2	4.7
**Preceding imaging (pre-PET/CT)**		
CT	29	67.4
MRI	1	2.3
PET/CT	3	7.0
Not documented	10	23.3
**Days between preceding imaging and PET/CT**		
Median	46	
Interquartile range (25–75)	20–117	

**Table 2:** Indications of PET/CT.

	No. of Patients (n = 43)	%
PET/CT indication		
Primary staging	2	4.7
Restaging	28	65.0
Evaluation residual tumor	16	37.2
Evaluation of treatment response	6	14.0
Evaluation of surgical resectability	4	9.3
Intermediate staging during therapy break	2	4.7
Assessment of dignity	13	30.2

**Table 3:** PET/CT findings.

	No. of Patients (n = 43)	%
**Metastases**		
Lymph nodes	36	83.7
Visceral organs	4	9.3
Lung/pleura	3	7.0
Bone	1	2.3
Brain	1	2.3
**Metastatic spread**		
1 organ	25	58.1
2–3 organs	5	11.6
>3 organs	2	4.7
**New secondary diagnosis**	4	9.3
Oncologic	1	2.3
Non-oncologic	3	7.0

**Table 4:** Changes in clinical stage after PET/CT according to histopathology and indication.

	Relapse	Upstaging	No Change	Downstaging	Not Clear
**Histopathology**					
**Testicular GCTs**					
Seminoma	4	4	15	6	1
Non-seminoma					
Embryonal carcinoma	0	0	2	2	0
Mixed GCT	0	2	3	0	0
**Extratesticular GCTs**					
Mediastinal seminoma	0	1	0	1	0
**Unknown**	1	0	0	1	0
**Indication**					
Evaluation of residual tumor	2	2	5	5	0
Evaluation of treatment response	0	0	3	2	0
Evaluation of surgical resectability	1	1	2	0	0
Intermediate staging during therapy break	0	0	1	1	0
Assessment of dignity	0	3	5	3	0

**Table 5:** The influence of PET/CT on the intended clinical management of patients.

	Before PET/CT		After PET/CT	
	n	%	n	%
**Intended treatment concept**				
Curative	26	60.5	26	60.5
Palliative	0	0	2	4.7
No information/unknown	17	39.5	15	34.9
**Intended mode of therapeutic intervention**				
Chemotherapy	3	7.0	11	25.6
Radiotherapy	1	2.3	2	4.7
Surgical resection	11	25.6	2	4.7
**Intended invasive diagnostics**				
Surgical biopsy	9	20.9	1	2.3
**Intended additional imaging**				
CT	22	51.2	3	7.0
MRI	5	11.6	0	0.0
Ultrasound	1	2.3	0	0.0

## Data Availability

The data presented in this study are available on request from the corresponding author. The data are not publicly available due to ethical restrictions.
